# The interactive reading task: Transformer-based automatic item generation

**DOI:** 10.3389/frai.2022.903077

**Published:** 2022-07-22

**Authors:** Yigal Attali, Andrew Runge, Geoffrey T. LaFlair, Kevin Yancey, Sarah Goodwin, Yena Park, Alina A. von Davier

**Affiliations:** Duolingo, Pittsburgh, PA, United States

**Keywords:** automatic item generation, reading assessment, language modeling, transformer models, psychometrics

## Abstract

Automatic item generation (AIG) has the potential to greatly expand the number of items for educational assessments, while simultaneously allowing for a more construct-driven approach to item development. However, the traditional item modeling approach in AIG is limited in scope to content areas that are relatively easy to model (such as math problems), and depends on highly skilled content experts to create each model. In this paper we describe the interactive reading task, a transformer-based deep language modeling approach for creating reading comprehension assessments. This approach allows a fully automated process for the creation of source passages together with a wide range of comprehension questions about the passages. The format of the questions allows automatic scoring of responses with high fidelity (e.g., selected response questions). We present the results of a large-scale pilot of the interactive reading task, with hundreds of passages and thousands of questions. These passages were administered as part of the practice test of the Duolingo English Test. Human review of the materials and psychometric analyses of test taker results demonstrate the feasibility of this approach for automatic creation of complex educational assessments.

## Introduction

### Automatic item generation

The advent of internet-based computerized assessment offers many advantages compared to more traditional paper-based assessments, including support for innovative item types and alternative item formats (Sireci and Zenisky, 2006), measurement of more complex knowledge, skills, and competencies (Bartram and Hambleton, 2005), automated scoring (Shermis and Burstein, 2013) which also allows immediate feedback to students (Attali and Powers, 2010), and adaptive testing and testing on-demand (van der Linden and Glas, 2010). These advantages have resulted in increased summative as well as formative testing and consequently the challenge of much higher volume of item development (Downing and Haladyna, 2006).

This challenge may be addressed through automatic item generation (Irvine and Kyllonen, 2002; Gierl and Haladyna, 2013). Automatic item generation (AIG) is usually based on the notion of an item “model” (Bejar, 2002), a schema or template for a question with parameters that can be instantiated with specific values. For example, the model X + Y = ?, where X and Y can be any whole numbers in the range 0–9, has two parameters. An item model can be instantiated, using a computer-based algorithm, to display an actual item (in this case, a single-digit addition exercise). A more complex example is “How many pieces of [fruit] will you have if you cut [5] whole [fruits] into [thirds]?” (Attali, 2018), where the text in parentheses represent parameters (numeric or text).

AIG has been used to create items in diverse content areas and formats (Haladyna, 2013). From a practical standpoint, the use of item models expands the potential number of items. From a theoretical standpoint, item models provide an opportunity for a construct-driven approach to item development (Embretson and Yang, 2006) because they can be tied to a mapping of the construct through an analysis of the cognitive mechanisms related to the item solution and item features that call on these mechanisms (Whitely, 1983).

However, an important limitation of the item model approach and its conventional implementation is that it is limited in scope to content areas that are relatively easy to model (such as math). In addition, because the process depends on highly skilled content experts to create the models, the AIG process is semi-automatic and can be costly in terms of the required resources.

As a result, the use of AIG as a technique to populate test or examination content is limited to relatively simple tasks. An example of a complex type of task that is not amenable to AIG is a reading comprehension task, which is the most common method used for assessment of higher-level verbal skills and abilities. Setting aside the difficulty of generating the reading passage, many reading comprehension questions would be unique for every passage, and therefore could not be produced from a simple item model. Other questions may have a standard stem (e.g., “What is the main idea of the passage?”), but the answer is of course unique to each reading passage, thereby shifting the burden to automatic scoring of possible responses (Shermis and Burstein, 2013).

A related literature in machine learning and natural language processing concerns automated question generation (AQG) for educational purposes. A recent review of this literature (Kurdi et al., 2020) classified AQG studies in terms of method of generation as template-based, rule-based, and statistical methods. Templates are a different term for models and are the most common approach used in the research reviewed. Rule-based approaches annotate sentences with syntactic and/or semantic information and then manipulate the input using suitable rules to create a question. For example, to create a WH question from a sentence, the rules would specify how to select a suitable question type (a suitable wh-word) and how to convert the sentence into a question. Template-based and rule-based methods similarly require manual development of the templates or rules (Kurdi et al., 2020). In contrast, with statistical methods the rules for question generation are implicitly learned through sophisticated language models.

### Modern language modeling

Language modeling seeks to develop a probabilistic model of language that can be subsequently used both to determine how likely a given text is and to stochastically generate plausible continuations to a given text. Language modeling has been at the core of natural language processing (NLP) research for decades (Shannon, 1951; Ney et al., 1994), but the most recent advances based on neural transformer architectures (Vaswani et al., 2017; Devlin et al., 2019; Radford et al., 2019) have made significant progress on the goal of being able to generate long, coherent, and information-rich sequences of text. What distinguishes transformer architectures from earlier neural network architectures is their ability to allow long-distance lexical relationships in text to have a much more direct influence on the task of predicting the next token in a sequence, allowing the model to more effectively leverage significantly longer contexts when making its predictions.

While these models often require a tremendous amount of training data and computing power to train initially, the trained models can be used to generate representations of language that can be subsequently used as inputs to train models for other language-related tasks such as text classification and question answering (Sun et al., 2019; Yang et al., 2019). These task-specific models typically need significantly less labeled training data in order to achieve the same or better results as systems trained on one or two orders of magnitude more data. However, they still require updating the model with a non-trivial amount of expert annotated data.

One of the most impressive recent examples has been OpenAI's GPT-3 model (Brown et al., 2020). This model has demonstrated the ability to learn the format and style of a text based on fewer than 10 examples in order to generate novel, coherent content without the need to explicitly update the underlying model. Because it mirrors the format as well as the style of any natural text, the generated output of the model can be natural text, enumerated lists, a paragraph paired with attributes or comprehension questions, or even well-formatted HTML code. As such, models such as GPT-3 represent a tremendous opportunity for innovations in AIG by allowing test designers to prototype and iterate on new item types without the need for significant expert-annotated data or lengthy model development and training processes.

#### Early implementations for AIG

von Davier (2018) successfully demonstrated that non-cognitive personality items can be generated by training a type of recurrent neural network known as long short-term memory (LSTM) network on a set of established personality statements. Hommel et al. (2021) used a transformer-based model to produce similar items.

Kumar et al. (2018) generated question-answer pairs from sentences using an LSTM. The answers are one of the named entities in the sentence and a model selects the most appropriate entity as the pivotal answer, around which a question is then generated. Kumar et al. (2019) present an extension of this work with a web-based interface for test developers to select and filter questions and answers generated from the model.

Sphinx (Khan et al., 2020) is a hybrid system that automatically generates reading texts using advanced language modeling techniques, but relies on human experts to generate reading comprehension questions, and item models to generate simple grammatical questions, such as sentence fragment correction questions (Khan et al., 2020, Figure 10).

## The interactive reading task

The interactive reading (IR) task is a framework for AIG and automatic scoring of reading comprehension (RC) passages and a suite of questions associated with the passage. The IR task requires test takers to sequentially interact with the text for several purposes that underpin the construct of reading (Grabe and Stoller, 2013). This task closely represents the activity of reading in a university setting and taps into established purposes for reading from the second language reading and language assessment research literature (Grabe and Jiang, 2013). The IR task complements the psycholinguistic approach of the Duolingo English Test on assessing reading comprehension by focusing more on the product and consequences of reading comprehension.

### IR question types

IR questions were designed to address several unique challenges:

- The questions should cover a wide range of RC component abilities and academic purposes for reading
- The questions should be able to support AIG and automated scoring
- The questions should be administered sequentially on the same text to use limited testing time in an efficient manner.

The following list provides an overview of the sequential IR questions:

- **Vocabulary in context (cloze)**. Only the first part of a text is presented, with several individual dispersed words blanked out throughout the text, and the test-taker is asked to complete the missing words.
- **Text completion**. The first part of the text is presented in full, together with the second part of the text. However, a sentence is missing between the two parts and the test-taker is asked to complete the missing sentence.
- **Comprehension questions**. The full text (two parts and missing sentence) is presented in full and the test-taker is asked to answer comprehension questions about the text.
- **Main idea**. The test-taker is asked to identify an idea presented in the text.
- **Possible title**. The test-taker is asked to identify the best possible title for the text.

For examples of IR passages with associated questions, see the Duolingo English Test guide for test takers (Duolingo, 2022) and the Duolingo English Test technical manual (Cardwell et al., 2022).

#### Construct coverage

The proposed questions cover major component abilities for reading comprehension (Grabe and Jiang, 2013).

- Vocabulary, morphological, and syntactic knowledge are addressed by the cloze question
- Text-structure awareness and discourse organization are addressed by the text completion and main-idea questions
- Main-idea comprehension is addressed by the main idea question
- Inferences about text information and summarization abilities are addressed by the possible title question
- Recall of relevant details and inferences about text information are addressed by the comprehension questions
- Fluency, rapid word recognition, and search processes are addressed by all questions.

These questions also address multiple academic purposes for reading (Grabe and Jiang, 2013): Reading to search for information (scanning and skimming), reading for quick understanding (skimming), reading to learn, reading to use information, and reading for general comprehension.

#### Question format and grading

The IR questions can be administered in multiple formats, including open-ended and selected-response formats. There is a sharp tradeoff associated with these two general formats. Open-ended questions are much easier to automatically generate (trivial for some of the questions) but very difficult to score automatically. For example, the c-rater (Leacock and Chodorow, 2003) short-answer automated-scoring engine requires hundreds of human-annotated training examples for each question, and as a result has been used mostly in research studies (Attali et al., 2008; Liu et al., 2016), as opposed to operational high-stakes assessments. The alternative selected-response format is trivial to score but can be very difficult to automatically generate the associated options, both distractors and correct answers. In general the IR questions were designed as selected-response questions and option generation is at the heart of the AIG approach outlined below.

One exception for the current set of questions is the comprehension questions that employ the format of text highlighting where the test taker is asked to highlight (click and drag to select) the answer in the text to the question. Although this format is open-ended, responses are compared to a single correct answer (a particular part of the text). For grading purposes, a text selection is defined as a point in the two-dimensional space for the location of the start and end indexes of the selection. A continuous grade between 0 and 1 is then calculated based on the discrepancy (geometric distance) between the point representations of the response and the correct answer. This grading approach is more nuanced than one which only considers the degree of overlap between a response and the correct answer. For example, two responses might both have no overlap with the correct answer, but one can be closer than the other to the correct answer (and therefore will be assigned a higher grade).

#### Future questions

In addition to the core questions outlined above, other IR questions are being developed, including questions that will address synthesis and critical reading skills. We are also developing questions with integrated modalities, such as reading-listening-writing (a question about the text is spoken, rather than presented in text) and reading-speaking (an answer to a question has to be spoken rather than typed).

### Passage and item generation

The IR AIG framework is based on the use of a Transformer-based language model to create texts and associated materials from which reading passages, questions, correct answers, distractors (for selected-response items), and other information necessary for automated scoring are extracted. For our experiments, we used the GPT-3 (Brown et al., 2020) family of models, which allow for few-shot conditioning of output without an explicit fine-tuning step.

#### Passage generation

We start by generating a source passage using a Transformer-based Language Model by providing to the model:

- A set of instructions, which are goals or general characteristics of the desired text output
- A set of examples for use in “seeding” the model

° Each example is a text and is associated with a desired format, subject matter, and style

- A set of attributes to control, or condition, the final characteristics of the text output.

The examples are aligned with a particular format, subject, narrative style, or other characteristics, which enables control over the qualities of the generated text. For example, the labels can be used to control the topic or domain of the text, the register, the format (i.e., dialogue vs. paragraph vs. enumerated list), allowing a flexible template for generating text with a range of qualities that could be useful for defining items with different goals. The instructions are a human-readable description of the type of content to generate, in some cases along with other basic details, such as “Generate short paragraphs from high school textbooks on the specified topic.”

#### Question and answer generation

We continue by generating questions and possible correct answers in a similar way to generating the source passage, by providing a set of instructions and examples (which consists of a set of passages and the desired questions and correct answers) together with the source passage to condition the final output of the model.

Specifically, for the main-idea and title tasks, the examples include a text and its associated main-idea or title. We generate multiple potential answers stochastically and evaluate them based on their similarity to the passage and average negative log likelihood as estimated by the language model. Similarity is computed by encoding the passage and each candidate answer using the SentenceTransformers^1^ library into a vector representation and computing the cosine similarity between them. We can also compute similarity between a candidate correct answer and each sentence in the passage to measure how well an answer aligns with a particular section of the passage. The average negative log likelihood is derived from the output distribution of the large language model. The probability of each token in a candidate answer, conditioned on all prior tokens, is estimated by the language model. These are then converted to negative log likelihoods and averaged over each token in the sentence to give a representation of how likely the answer is, based on the examples the model has seen.

For comprehension questions, examples consist of passages with multiple potential questions and their associated answers. The model is then prompted to generate new questions and their answers for a source passage. To better guarantee that the generated questions are answerable using the passage, we use an external question answering model^2^ to predict the overall likelihood that the question can be answered. We filter out questions with a low answerability likelihood (determined through experimentation and manual review), questions that are extremely long (>25 words), questions with answers that are very short (1–2 words), and questions whose predicted answers do not align well with text in the passage.

For the completion task, we identify candidate target sentences by estimating the likelihood of each sentence in the text, averaged over the tokens in the sentence, using the language model. Candidates must have between 8 and 30 words and cannot be one of the first two sentences or the last sentence in the passage. The best candidates will have high likelihood, given the first part of the passage. In addition, the first few sentences immediately following the candidate should also have a high likelihood, indicating that the candidate sentence fits in well with both its preceding and following contexts.

To generate a set of possible incorrect answers (distractors) for the main-idea and title selected-response tasks, we generate alternative texts in addition to the original main source passage. These alternative texts are generated using the same instructions, examples, and conditioning attributes as the original text, thereby rendering them stylistically and topically similar to the source passage, but they differ in the exact content, making them ideal for use in generating incorrect answers for questions to the original source passage. Accordingly, for each of the alternative passages, we generate a set of possible answers to each item using the same instructions and examples for generating possible correct answers, but conditioning on the *alternative passage* instead of the original one. An example of automatically produced alternative texts, as well as the main ideas and titles associated with them, is presented in the Appendix. The main ideas and titles for the original text would serve as the correct answers for the corresponding tasks, whereas those for the alternative texts would be used as distractors for the corresponding tasks.

Lastly, we compute a suite of NLP metrics for the pool of correct and incorrect option candidates in order to select a single correct answer and several incorrect answers for the item from these candidates. These include the average similarity of the answer to other correct answer candidates, similarity of the answer to the source passage and to individual sentences in the source passage, and the model's estimated probability of generating the candidate answer.

#### Vocabulary in context

A different process is used for generating vocabulary in context items. To select words for deletion, the language model is used to iteratively complete each word in the source passage. The model computes likelihoods for each word in its vocabulary. Candidate words for deletion are then filtered based on the likelihood and rank order of the original word being suggested by the model (e.g., a word is more likely to be selected if it has the highest likelihood), syntactic information about the word (e.g., nouns are more likely to be selected than adjectives), semantic information about the word (e.g., the frequency of the word is taken into account in the context of passage difficulty), and the distance (i.e., the number of words that separate consecutive deleted words) between the original word and the nearby successful candidates (e.g., the further a word is from other candidates, the more likely the word is to be selected for deletion). We use Spacy (Honnibal et al., 2020) for part of speech tagging and lemmatization and the Corpus of Contemporary American English (Davies, 2009) for word frequencies. Candidate distractors for deleted words are then selected from the model's likelihood output for all other words in its vocabulary. Ideally, successful distractors have low, but not too low, likelihood, and have the same syntactic part-of-speech as the correct answer.

### Human review

All materials, including source passages, questions, correct answers, and distractors, are reviewed by subject-matter experts (SME) with expertise in reading comprehension test development. These SMEs take into account editorial and test design considerations in reviewing the materials. Since most passages successfully pass this review process, any passage that is deemed to require more than a few minor edits is discarded. Lastly, all final materials go through fairness and bias review by a separate group of reviewers.

## Large-scale pilot

The IR task was developed through an iterative process that included multiple pre-pilots that examined task design and psychometric issues. As such, this process exemplifies the computational psychometrics approach (von Davier et al., 2021), which blends data- driven computer science methods (machine learning and data mining, in particular) and theory-driven psychometrics and language assessment considerations in order to measure latent abilities in real time. This blend is often instantiated as an iterative and adaptive process (von Davier, 2017). The culmination of this process was a large-scale pilot, described in this section, whose purpose was to evaluate the quality of the AIG processes described above, both from a human review and psychometric perspective.

### The Duolingo English test

The development and large-scale pilot of the IR task was carried out in the context of the Duolingo English Test (DET). The DET is a digital-first English language proficiency assessment that is used by colleges and universities to make admissions decisions. A digital-first assessment is an assessment that has been designed to be digital end-to-end, with automatic tools and theoretical frameworks fluidly integrated for an optimal test taking experience (they are in contrast to digitized assessments that represent traditional assessments that have been moved online). A key consideration in the design, development, administration, and scoring of the test is the test taker experience (Burstein et al., 2021). The test is administered to test takers in the Duolingo English Test Desktop App *via* the internet and can be taken anytime anywhere in the world. Furthermore, the test leverages advances in machine learning and computational psychometrics to create, score items, and administer them adaptively. Its availability and 1-h duration creates an improved experience for test takers over other standardized tests, which take 3–4 h to complete and can only be taken at a testing center, sometimes hundreds of miles away from the test taker's home. Test scores are reported within 48 h of test completion, and test takers can share their scores with as many accepting institutions as they need, free of charge (Cardwell et al., 2022).

### Initial machine-generation phase

As an initial step, over 14,000 passages were generated in three text genres: news, expository, and narrative texts. We provided 3–5 examples to GPT-3 of the given genre, with each example consisting of a topic, title, and passage. When generating new passages, we conditioned the output by just the topic, with expository and narrative texts generated based on 270 possible topics corresponding to common fields of university study, while news texts were based on 45 common news article categories.

This set of passages went through two rounds of automated filters. The first set filtered the passage based on general desirable qualities of the text. We filtered passages to be 100–175 words with 5–20 sentences. We removed passages that contain repeated 8-grams to avoid passages containing a high degree of repetition by the language model, as well as passages that contain profanity or other potentially offensive content using a standard offensive word list. We also filtered passages in two ways based on the negative log likelihood of each sentence as estimated by the language model, averaged over the tokens: We imposed an upper bound on the maximum negative log likelihood that any sentence can have as a simple estimate of the expected coherence of the passage. If a sentence has extremely high negative log likelihood, it is more likely to make less sense in the context of the passage. Additionally, for the completion task we imposed an even stricter negative log likelihood threshold to identify viable candidate sentences, as we wanted to ensure that the correct answer is very likely to fit in the context of the passage. These thresholds were identified through manual review of passages and potential candidate sentences.

For this pilot effort, we sampled 800 texts from the set of passages that passed this first round of filters. We then generated items for these texts and simultaneously filtered them based on the ability of our item generation processes to generate appropriate items with sufficient distractors according to the metric-based criteria described in the previous section. Passages for which items and distractors could not be generated according to our specifications were removed from our set. We retained a set of 789 passages following the item generation and filtering process. For each of these passages we generated a vocabulary-in-context task (with 6.6 fill-in-the-blank words on average, and with four distractors), one text completion task, two comprehension tasks, one main-idea task, and one possible title task. All selected-response tasks (except for the vocabulary-in-context task) were generated with three distractors.

### Human review

Content and fairness review was conducted by 12 external reviewers and six internal Duolingo team members. External reviewers had diverse backgrounds with regard to gender identity, age, and racial/ethnic background. All had at least a bachelor's degree (and in some cases a Ph.D.) in linguistics, language studies, or a related field. They had expertise in teaching and assessing in a relevant linguistic and cultural context. Internal team members had in most cases a Ph.D. and considerable expertise in assessment development.

Each passage and question went through multiple rounds of reviews, with a minimum of three content reviews and two fairness reviews. Content reviewers independently evaluated the appropriateness of the content and made edit suggestions. For passages, content reviewers evaluated the cohesion, clarity, and logical consistency throughout the text. For questions, reviewers judged the viability of each option by ensuring that the correct answer is correct and the distractors are incorrect. In cases where the evaluation recommended hefty edits to the passage or the questions, these edits were reviewed, adjudicated, and incorporated by additional reviewers.

Following assessment fairness guidelines (Zieky, 2015), fairness reviewers reviewed passages and questions to ensure they did not contain any content that was too culturally specific, had technical or field-specific jargon, or could be potentially sensitive to test takers.

In summary, following all reviews and adjudication a final set of 454 out of 789 passages (58%) were retained. The review process is estimated to have taken about 15 min per passage (including questions), across all rounds of review.

### Administration

The pilot for the IR task was administered at the end of the DET practice test (https://englishtest.duolingo.com/home), which is a shorter version of the operational DET and is freely available. Similar to the operational test, the practice test is fully adaptive and test takers have the opportunity to respond to and practice all of the tasks that are included in the operational test. At the end of the practice test, test takers were randomly assigned one of the 454 passages. The time limit for the IR section was 8 min (determined based on previous pilots). The pilot was active for 21 days, during which nearly 200 thousand IR sessions were completed.

### Psychometric results

This section summarizes results from various psychometric item analyses. As a reminder, the pilot included 454 passages, each with a vocabulary-in-context task (with 6.6 fill-in-the-blank words on average), one text completion task, two comprehension tasks, one main-idea task, and one possible title task. A total of 5,246 items (the term item is used in this section to refer to a measurement opportunity) were fielded with an average of 425 responses collected per item.

#### Response time

**Figure 1:**
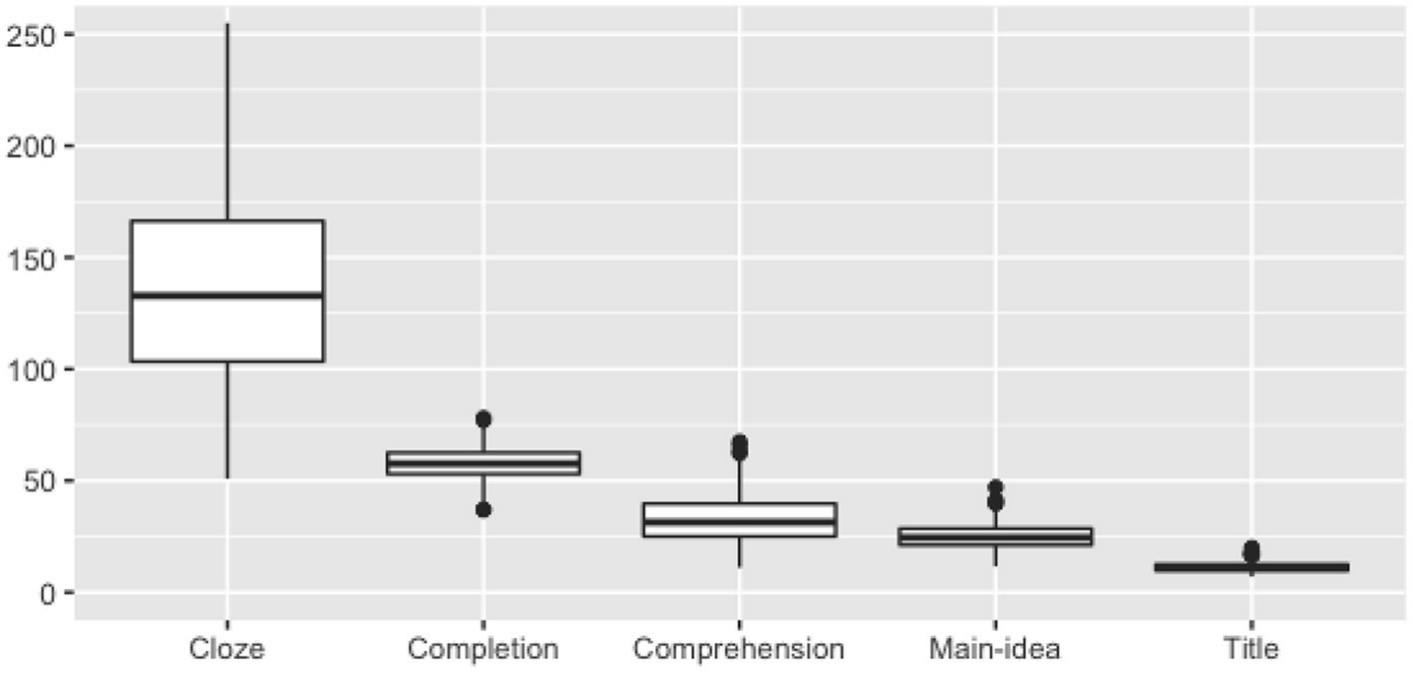
Median response time distributions (in seconds).

One of the purposes of the interactive nature of IR passages is to take advantage of earlier processing of the text to reduce the time required for answering later questions. As expected from this design characteristic of the task, Figure 1 shows a sharp decrease in response time from the first task (cloze) to later tasks.

#### Easiness and discrimination

The two primary psychometric indicators for items are item easiness and item discrimination. Item easiness (or difficulty), measured here as the mean score on the item (which is equal to percent correct for all tasks except the comprehension task), evaluates the degree that test takers successfully respond to the items. Item discrimination evaluates how well an item is able to distinguish between test takers who are knowledgeable and those who are not. Item discrimination is measured here with item-total correlations, where total was defined as the total practice test score. A higher item-total correlation signifies that test takers with higher total scores were more likely to also answer the item correctly.

**Figure 2:**
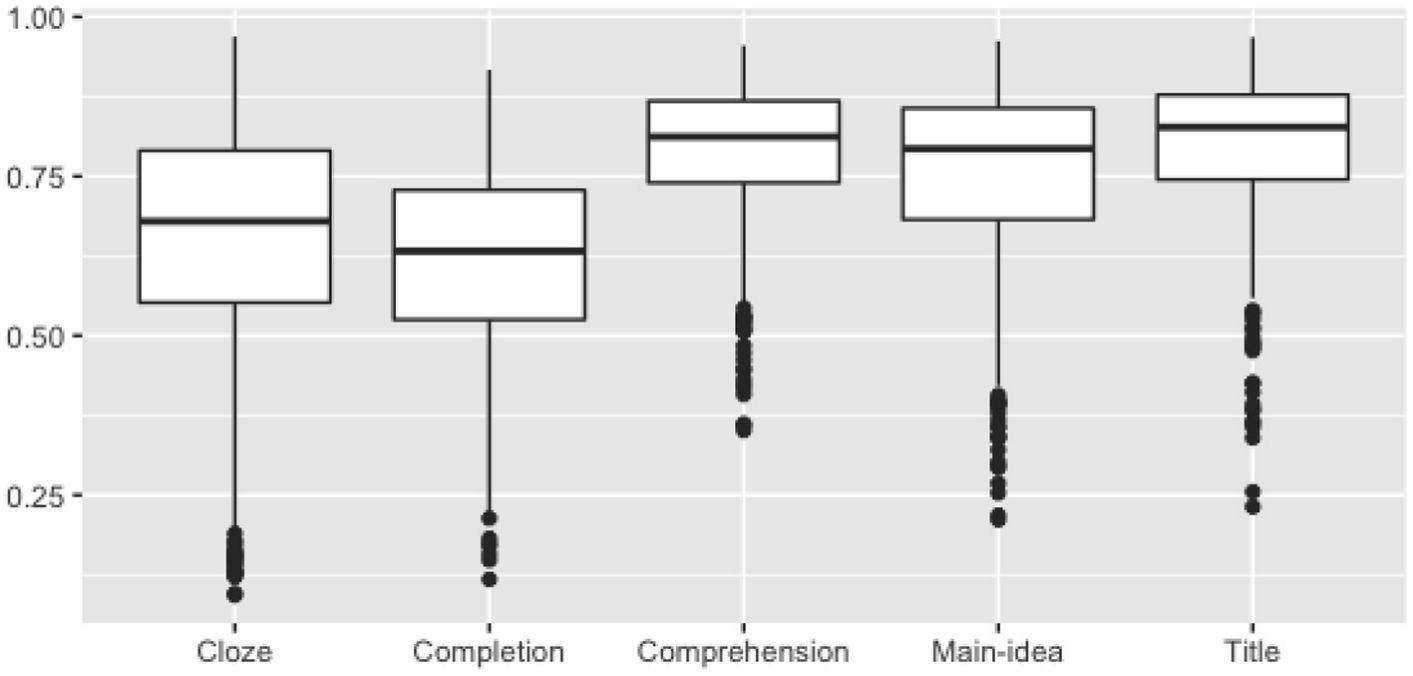
Mean score (easiness) distributions.

Figure 2 shows a wide distribution of item easiness (with an overall mean score of 70%) with earlier tasks (cloze and completion) less easy than later tasks.

**Figure 3:**
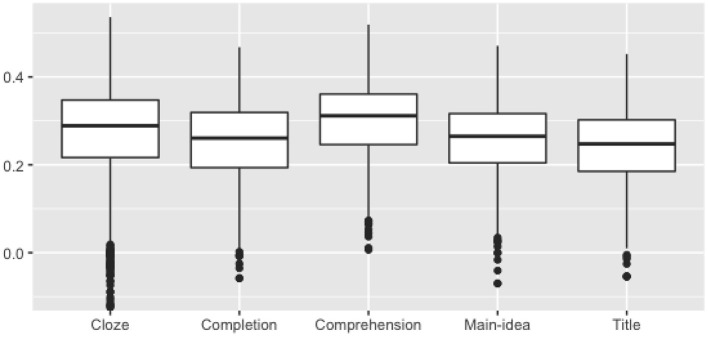
Item-total correlations with total practice test score.

Figure 3 presents distributions of item-total correlations. Note that the practice test does not include reading items and is also quite short (up to 15 min), both of which would have the effect of moderating item-total correlations. Nevertheless, most items show reasonably high discrimination (with an overall average of 0.27), with small differences across tasks. In total, only 6% of items had an item-total correlation lower than 0.1. However, even this number can be significantly reduced through a distractor analysis. By computing discrimination indices for each distractor (see Attali and Fraenkel, 2000) we can identify distractors for which the average total score of test takers endorsing them is higher than the average total score of test takers endorsing the correct answer. About 3% of all distractors are failing in this way. Removing these distractors (essentially administering the item with one less distractor) reduces the number of items with item-total correlation lower than 0.1 to only 2%.

#### Local item dependence

An important assumption for psychometric analysis of test items is that the dependency between responses to any pair of items is due only to the trait being measured. Pairs of items that violate this assumption are said to exhibit local item dependency (LID). It is well-known (Yen, 1993) that sets of items that are based on a common stimulus, such as reading comprehension questions, can result in local dependence that is due to the fact that information used to answer different items is interrelated in the stimulus. In the present context, the threat of LID is even greater since the items were automatically generated. In particular, we expected that LID is more likely to be present between two deleted words of the same cloze task, as well as between two later tasks (e.g., the main-idea and the title task).

A standard item response theory (IRT) approach for investigating LID between test items is to compute the correlation between residual responses—the difference between the expected model-based score and the actual observed score (Yen, 1984). As an approximation of this approach, we computed the partial correlations between pairs of items, controlling for total practice test score. In the analyses below, a common threshold of 0.3 (Christensen et al., 2017) was employed for categorizing residual correlations as indicating LID.

**Figure 4:**
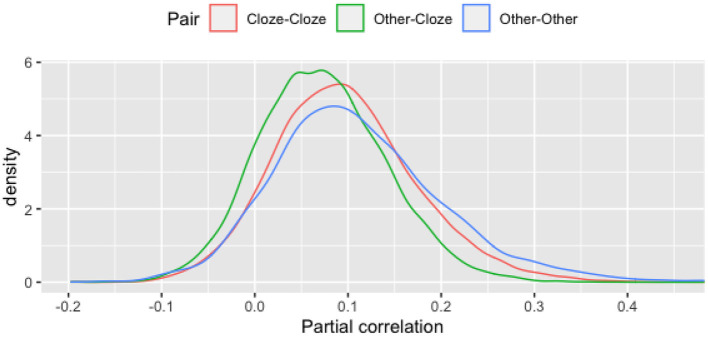
Partial correlations between item pairs.

Figure 4 presents distributions of these partial correlations for the three types of pairs of items. Although pairs of items that are not of the same type (Other-Cloze) have slightly lower partial correlations (with an average of 0.07) than either Cloze-Cloze or Other-Other pairs of items (0.1 and 0.11, respectively), only 1% of all 9,437 Cloze-Cloze pairs and 3% of all 4,540 Other-Other pairs exceed the threshold of 0.3.

## Future directions

### L2 vs. L1

The IR task can be used to assess reading skills of students from a variety of backgrounds and levels. However, in our study we focused on readers whose first language is not English. The second language (L2) readers who participated in this study are different from first-language (L1) readers of English in many respects, but also share some similarities, including cognitive and linguistic components such as word recognition, vocabulary knowledge, morphosyntactic knowledge, and world knowledge (Grabe and Jiang, 2013; Nassaji, 2014). The two groups are also similar in their advanced academic and professional jr reading strategies (Grabe and Stoller, 2013). In addition, since both groups are diverse and need to be able to read for a variety of different purposes, the IR task is potentially useful for both L1 and L2 reading assessment. This can be the focus of future research.

### Interactive assessment

Providing feedback regarding task performance is one of the most frequently applied of all psychological interventions (Kulhavy and Stock, 1989; Shute, 2008). Feedback helps learners determine performance expectations, judge their level of understanding, and become aware of misconceptions. It may also provide clues about the best approaches for correcting mistakes and improving performance. However, feedback has had almost no place in large-scale assessment (Attali and Powers, 2010; Attali, 2015).

The IR task has already incorporated interactivity with the gradual revelation of the passage: rather than presenting the passage in its entirety, the passage is sequentially revealed across three different item types. At the same time, a subtle form of corrective feedback is provided, whereby along with the rest of the passage, the answers to the previous items are also revealed. We are exploring ways to enhance feedback. For example, test takers could receive immediate feedback about the correctness of their responses and be asked to try again in the case of errors. This type of multiple-try feedback has been shown to decrease test anxiety and improve measurement accuracy by allowing partial credit grading (Attali and Powers, 2010; Attali, 2011).

### Modeling of task difficulty

Our methods for passage and item generation allow us to generate texts with a range of difficulty levels by controlling the register and domain of the generated text. Much prior work has explored ways to estimate the difficulty of reading passages and comprehension questions based on a wide range of lexical, syntactic, and discourse properties (Xia et al., 2016; Huang et al., 2017; Settles et al., 2020). Additionally, recent work has demonstrated the ability to estimate the psychometric properties of passage-based items using a featurized approach based on the BERT transformer model (Devlin et al., 2019), allowing for the estimation of parameters of newly created items that do not yet have any response data (McCarthy et al., 2021). This work primarily focuses on estimating the psychometric properties of items that test vocabulary in context, so we plan to explore methods to adapt it to multiple choice and reading comprehension questions about the passages. These estimates can then be used to refine our item generation processes in order to select passages, distractors and comprehension questions that will result in the items having the desired psychometric properties.

### Psychometric modeling

The current set of IR questions include a mix of binary graded questions (for selected-response questions) and continuous graded questions (between 0 and 1, for highlight comprehension questions). This presents unique challenges in terms of psychometric modeling, since continuous response models are not often discussed in the context of IRT. For modeling responses on the unit interval, the Beta distribution has been proposed in the psychometrics literature (Noel and Dauvier, 2007; Chen et al., 2019). However, existing approaches are limited in different ways. The approach by Chen et al. (2019) assumes that latent ability is constrained to the unit interval, a constraint that is inconsistent with normal IRT assumptions. The approach by Noel and Dauvier (2007) does not include discrimination parameters, a constraint that is also inconsistent with other common IRT models, such as the 2PL model. The IR task would require innovative psychometric models that can combine discrete and continuous grades, as well as support modeling of item discrimination.

### Other types of texts and tasks

The IR task is a rich breeding ground for more innovations in terms of both assessment design and content generation. Other types of texts can be considered as candidates for passage generation; for instance, argumentative texts of multiple viewpoints would support additional tasks like synthesis. Another possible avenue is different response formats. The digital format of IR makes it possible to accommodate multiple types of response formats, such as dragging and dropping, free response (both spoken and written), and interacting with other media such as a chart, a table, or a graph. The advantage of the digital format can be extended to the modality of the delivery as well, where, for instance, comprehension questions could be delivered aurally. All of these would allow for a fuller approximation of what is read, how it is read, and what comes after reading in academic contexts.

## Conclusions

This paper demonstrates how recent advances in computational language modeling can transform item development for complex tasks and assessments. A combination of task design, text generation techniques, and psychometric analysis allows us to create reading passages and associated assessment tasks that can be automatically scored. This has not been possible with more traditional AIG approaches. These advances in turn support increasingly complex digital-first assessment systems that integrate a theoretical framework of domain expertise with AI tools, technology infrastructure and psychometrics.

## Data availability statement

The original contributions presented in the study are included in the article/supplementary materials, further inquiries can be directed to the corresponding author.

## Ethics statement

Ethical review and approval was not required for the study on human participants in accordance with the local legislation and institutional requirements. The patients/participants provided their written informed consent to participate in this study.

## Author contributions

All authors listed have made a substantial, direct, and intellectual contribution to the work and approved it for publication.

## Conflict of interest

YA, AR, GTL, KY, SG, YP, and AAD were employed by Duolingo.

## Publisher's note

All claims expressed in this article are solely those of the authors and do not necessarily represent those of their affiliated organizations, or those of the publisher, the editors and the reviewers. Any product that may be evaluated in this article, or claim that may be made by its manufacturer, is not guaranteed or endorsed by the publisher.

## Footnotes

^1^ https://www.sbert.net/

^2^ https://huggingface.co/deepset/roberta-base-squad2

## Appendix

### An example of alternative texts

#### Original text

From the largest animals to the tiniest, an ecosystem can be organized by energy into a pyramid called the energy pyramid. At the bottom of the pyramid are producers, which are organisms that make their own food, such as plants. Plants produce food by taking in carbon dioxide from the air and water from the soil. Plants use sunlight to create sugars from the carbon dioxide molecules and water molecules. The plant moves sugars through its body and assembles these sugars into long chains of molecules called polysaccharides. The polysaccharides may be used immediately, or stored for later use by the plant. A plant may store several million calories of energy in this manner, enough to live for several weeks or more without sun. Most of this energy is glucose, a sugar that animals also need to survive. The rest of the organisms in the pyramid rely on the energy that comes from producers.

**Main idea**: Producers are at the bottom of the energy pyramid and are the source of food and energy for the other organisms in the ecosystem.

**Title**: Producers in an Ecosystem

**Comprehension Questions:** What do plants use to create energy? How can a plant survive for weeks without sunlight?

#### Alternative text 1

In biology, the term energy pyramid refers to a graphical way of representing the position of energy-storing or energy-providing organisms in an ecosystem. Organisms on the lower levels of the pyramid—primary producers—can make their own food from sunlight and inorganic substances, while those on the upper levels—consumers—gain their nutrition from other living things. The energy pyramid also shows how each level is dependent on the one below it. The food chain shows how energy from the sun is passed along from one organism to another, where it is eventually lost when organisms die, decompose, or are eaten.

**Main idea**: The energy pyramid is a graphical representation of how energy is passed along from one organism to another in an ecosystem.

**Title**: Energy Pyramids in Ecosystems

#### Alternative text 2

The energy pyramid is a model that shows the flow of energy in an ecosystem. The sun is the source of all energy in the ecosystem, and plants use this energy to create food. The energy then flows from the plants to the animals that eat them. When animals eat plants, they only use a small amount of the energy that the plants have stored. The rest of the energy is lost as heat. This is why there are fewer animals at each level of the food chain. When animals eat other animals, they also use only a small amount of the energy from the animal they are eating. The vast majority of the sun's energy is lost as heat at each level of the food chain.

**Main idea**: The energy pyramid is a model that shows how energy flows from the sun to plants to animals, with most of the energy being lost as heat at each level.

**Title**: Energy and the Food Chain
